# β3‐AR/β‐arrestin2 Interaction Triggers Cardiac Fibrosis Through JNK/c‐Jun Pathway in Cardiac Fibroblasts

**DOI:** 10.1111/jcmm.71305

**Published:** 2026-08-03

**Authors:** Zhongcheng Xu, Min Zhu, Chun Zhou, Juncang Duan, Jian Ding, Jun Liu, Xuan Ying, Jie Wu, Yang Dong, Wenbiao Wang, Zhaohui Qiu

**Affiliations:** ^1^ Department of Cardiology The Jinhua Affiliated Hospital of Wenzhou Medical University Jinhua Zhejiang China; ^2^ Department of Cardiology, Shanghai Tong Ren Hospital Shanghai Jiao Tong University School of Medicine Shanghai China; ^3^ State Key Laboratory of Cardiovascular Disease, Key Laboratory of Pluripotent Stem Cells in Cardiac Repair and Regeneration, Fuwai Hospital, National Center for Cardiovascular Diseases Chinese Academy of Medical Sciences and Peking Union Medical College Beijing China; ^4^ Department of Ophthalmology The Jinhua Affiliated Hospital of Wenzhou Medical University Jinhua Zhejiang China; ^5^ Department of Cardiology, Affiliated Jinhua Hospital Zhejiang University School of Medicine Jinhua Zhejiang China; ^6^ Department of Ultrasonography The Jinhua Affiliated Hospital of Wenzhou Medical University Jinhua Zhejiang China

**Keywords:** biased activation, cardiac fibrosis, JNK/c‐Jun pathway, β3‐adrenergic receptor, β‐arrestin2

## Abstract

Myocardial fibrosis (MF) is a critical pathological substrate of heart failure (HF), and β3‐adrenergic receptor (β3‐AR) has been implicated in cardiac remodelling with controversial roles. This study aimed to clarify the pro‐fibrotic mechanism of β3‐AR in cardiac fibroblasts and its association with β‐arrestin2‐mediated biased activation. Primary cardiac fibroblasts were isolated from neonatal C57BL/6 mice and subjected to β3‐AR overexpression, agonist (BRL37344) stimulation, antagonist (SR59230A) inhibition, JNK inhibitor (SP600125) treatment, or β‐arrestin2 knockdown via siRNA. RNA sequencing, immunofluorescence, Western blotting, and coimmunoprecipitation assays were performed to explore molecular mechanisms. RNA sequencing identified 577 upregulated and 231 downregulated genes in β3‐AR‐activated fibroblasts, enriched in extracellular matrix organisation and inflammatory pathways. Functional experiments confirmed that BRL37344 significantly upregulated fibrosis markers (α‐SMA, FN, collagen I/III) and TGF‐β1 expression, which was reversed by SP600125. Notably, inhibition of Gi signalling by pertussis toxin (PTX) failed to block β3‐AR‐induced fibrosis, while β‐arrestin2 knockdown abrogated JNK/c‐Jun/TGF‐β1 pathway activation. Immunofluorescence and coimmunoprecipitation verified colocalisation and direct interaction between β3‐AR and β‐arrestin2, which was enhanced by BRL37344. Our findings demonstrate that β3‐AR promotes cardiac fibrosis through β‐arrestin2‐mediated biased activation of the JNK/c‐Jun/TGF‐β1 pathway, independent of classical Gi signalling. This study reveals a novel cell‐specific signalling mechanism of β3‐AR and provides a potential therapeutic target for developing precision drugs against MF and HF.

## 1 Introduction

Myocardial fibrosis (MF) is a pivotal pathological driver of heart failure (HF) resulting from various cardiac diseases. It is characterised by abnormal deposition of extracellular matrix and disorganisation of perivascular collagen, leading to impaired myocardial stiffness, contractility, and coronary reserve [1]. The sympathetic nervous system (SNS) plays a central role in the neurohumoral regulation of MF: Sustained SNS activation elevates catecholamine levels, alters myocardial β‐adrenergic receptor (β‐AR) signalling, and mediates cardiac effects—making β‐blockers a cornerstone therapy for inhibiting MF and adverse remodelling [2, 3]. Among cardiac β‐AR subtypes, β1‐AR undergoes desensitisation and downregulation in HF, while β2‐AR shifts its coupling from Gs to Gi protein signalling. In contrast, β3‐AR—a later‐identified GPCR with seven transmembrane domains—is upregulated, suggesting a critical yet incompletely defined role [4, 5]. Initially cloned from human adipocytes in 1989 [6], β3‐AR has since been implicated in glucose/lipid metabolism and cardiac regulation. Early studies proposed that β3‐AR resists desensitisation due to the absence of canonical PKA/GRK phosphorylation sites; however, recent evidence indicates that GRK2 mediates its short‐term desensitisation, although the underlying mechanism remains unclear [7, 8].

The cardiac role of β3‐AR is supported by conflicting evidence. Niu et al. [9] demonstrated that β3‐AR agonists reduced MF, apoptosis, and ventricular dilation while improving cardiac function in C57BL/6J mice following myocardial infarction (MI), with similar cardioprotection observed in ischemia/reperfusion injury models [10]. In contrast, some studies reported that β3‐AR upregulation in rapid pacing‐induced atrial fibrillation (AF) models exacerbated atrial apoptosis, fibrosis, structural remodelling, and AF inducibility, while reducing phosphocreatine levels [11, 12]. These discrepancies may arise from the diverse activation modes of β3‐AR as a GPCR. Beyond canonical G protein coupling, GPCRs can engage β‐arrestin‐mediated biased signalling, whereby β‐arrestin binds phosphorylated β‐ARs to activate alternative pathways such as JNK3/ERK [13, 14, 15].

Biased agonism may overcome limitations of current HF therapies, which often cause severe side effects due to broad β‐AR blockade. For instance, biased opioid receptor agonists activate Gi signalling while attenuating β‐arrestin recruitment, reducing risks of addiction and respiratory depression [16]. Carvedilol, a β‐blocker that preserves β‐arrestin signalling, demonstrates superior clinical efficacy [17, 18]. Similarly, the β2‐AR‐biased agonist fenoterol enhances cardiac contractility via Gs signalling in canine HF models [19]. Our previous work revealed that excessive β3‐AR activation exacerbates MF through JNK/c‐Jun‐mediated upregulation of TGF‐β1 [20, 21]—contrasting with its conventional Gi‐coupled cardioprotective role—suggesting the involvement of alternative activation mechanisms such as β‐arrestin2‐mediated biased signalling. Supporting this, β‐arrestin2 knockdown suppressed TGF‐β1‐induced fibrosis in a rat MI model [22], GRK2/β‐arrestin signalling promoted cardiac fibroblast proliferation [23], and β‐arrestin2 was essential for metabolic effects of β3‐AR [24].

To verify our previous hypothesis, we will isolate primary cardiac fibroblasts and evaluate the pro‐fibrotic effect of β3‐adrenergic receptor (β3‐AR) via β3‐AR overexpression, along with the administration of its specific agonist and antagonist. Moreover, we will intervene in β‐arrestin2 expression using β‐arrestin2‐siRNA to investigate whether the pro‐fibrotic effect of β3‐AR is dependent on β‐arrestin2 rather than Gi protein, as well as the underlying downstream molecular signalling pathways. This study aims to provide a theoretical basis and potential therapeutic targets for the development of low‐toxicity, pathway‐selective precision drugs against MF and HF, thereby addressing the urgent clinical demand for safer therapeutic strategies for HF.

## 2 Materials and Methods

### 2.1 Isolation of Neonatal Mouse Cardiac Fibroblasts (NMCFs)

NMCFs are a well‐recognised model for cardiac fibrosis research due to their high viability, stable proliferation, high transfection efficiency, and conserved fibrotic signalling responses compared with adult cardiac fibroblasts [25, 26]. NMCFs were isolated and cultured as previously described [27]. In brief, the hearts of 1–3‐day‐old C57BL/6 mice obtained from SPF (Beijing) Biotechnology were digested with a mixed digestive solution including 0.08% trypsin (27250–018, Gibco, USA) and 0.06% collagenase II (17101–015, Gibco, USA) at 37°C. The isolated cells were plated in a T‐25 culture flask in Dulbecco's modified Eagle's medium (DMEM, Gibco) with 15% fetal bovine serum (FBS), 1% penicillin/streptomycin, and bromodeoxyuridine (BrdU, 100 μM) for 2 h to allow NMCFs to attach. The unattached cardiomyocytes were transferred to other dishes. The NMCFs were cultured at 37°C in humidified air containing 5% CO_2_ for subsequent treatment.

### 2.2 Cell Culture and Treatments

The NMCFs were cultured in DMEM with 15% FBS, 1% penicillin/streptomycin, and maintained at 37°C in a 5% CO_2_ atmosphere. To inhibit the activity of the Gi signalling pathway, NMCFs were pre‐incubated with pertussis toxin (PTX, #516560, Sigma, USA), followed by transfection with Adv‐Adrb3 (overexpressing β3‐AR) (MOI = 12.5) for 72 h. In the intervention experiment, NMCFs were treated with complete medium containing 10 μM BRL37344 (β3‐AR agonist, B169, Sigma, USA), or 10 μM SR59230A (β3‐AR inhibitor, S8688, Sigma, USA), or 10 μM SP600125 (JNK inhibitor, S1460, Selleck, USA) for 72 h. To interfere with the expression of β‐arrestin2 in NMCFs, the specific small interference RNA (siRNA, 5′‐UCUACAUUCACUCCUUGCGUU‐3′) of β‐arrestin2 was transfected into cells for 48 h, followed by treatment with 10 μM BRL37344 or 10 μM SR59230A for 72 h.

### 2.3 Immunofluorescence Staining

Mouse cardiac fibroblasts were seeded on a confocal dish and then treated with β3‐AR overexpression, BRL37344, or SR59230A. The cells were fixed with 4% paraformaldehyde for 10 min, followed by washing with phosphate‐buffered saline (PBS) for 5 min each time, repeated 3 times. The cells were treated with 0.3% Triton X‐100 in PBS for 15 min and then blocked with 5% bovine serum albumin (BSA) at room temperature for 1 h. The cells were then stained with the cardiac fibroblast marker Vimentin (1:100, sc‐32,322, Santa Cruz, USA), primary antibodies against β3‐AR (1:100, sc‐515,763, Santa Cruz, USA), β‐arrestin2 (1:100, #3857, CST, USA) at 4°C overnight, followed by incubation with a secondary antibody (1:500, ab150115 or ab150077, Abcam, UK) for 1 h, then the nuclear dye 4′,6‐diamidino‐2‐phenylindole (DAPI) for 10 min at room temperature. After the cells were washed three times, the confocal dishes were visualised under a fluorescence microscope (Leica, Germany).

### 2.4 RNA Extraction and Real‐Time RT–PCR

Total RNA was isolated from cells using TRIzol reagent (#15596026, Invitrogen, USA). One microgram of isolated RNA was reverse‐transcribed into cDNA using the TransGen reverse transcription Kit (AE301‐02, TransGen, Beijing, China). Quantitative real‐time PCR analysis was performed using PowerUp SYBR Green Master Mix (A25779, Thermo Fisher, USA) and a QuantStudio 3 Real‐time PCR system (Thermo Fisher, USA), and the expression of the β‐actin (β‐actin, ACTB) gene was used as an internal control. All primers used in this study are described in Table S1.

### 2.5 Western Blotting

Proteins from NMCFs were lysed using RIPA lysis buffer containing PMSF and a protease inhibitor cocktail. Protein (~50 μg) was separated via 10% sodium dodecyl sulphate–polyacrylamide gel electrophoresis (SDS–PAGE) and transferred to polyvinylidene fluoride (PVDF) membranes. The membranes were blocked with 5% non‐fat milk, incubated with primary antibodies overnight at 4°C, and then incubated with secondary antibodies for 1 h at room temperature. The bands were visualised with ECL reagents and quantified using a chemiluminescence imaging system. The primary antibodies used for western blotting in this study were against p‐JNK (1:2000, #9255, CST, USA), JNK (1:1000, #9258, CST, USA), p‐c‐Jun (1:1000, #3270, CST, USA), c‐Jun (1:1000, #9165, CST, USA), β‐arrestin2 (1:1000, #3857, CST, USA), TGF‐β1 (1:500, sc‐130,348, Santa Cruz, USA), and β‐actin (1:10000, 66,009–1‐Ig, Proteintech Group, USA).

### 2.6 Coimmunoprecipitation (Co‐IP) Assays

Cells were collected and rinsed twice with pre‐cooled PBS, then lysed in 500 μL of RIPA lysis buffer containing protease inhibitors and PMSF. The cell lysis was incubated with anti‐β‐arrestin2 antibody or IgG on a shaker at 4°C for 6 h; then protein A agarose beads were added to the mixture and incubated overnight on a shaker at 4°C. After the co‐immunoprecipitation reaction, centrifugation was performed at 3,000 rpm for 3 min at 4°C to pellet the agarose beads at the bottom of the tube; the supernatant was carefully aspirated, and the agarose beads were washed 3 times with 1 mL of lysis buffer. Finally, 15 μL of 2 × SDS loading buffer was added, and the mixture was boiled in water for 5 min, followed by SDS–PAGE and Western blotting analysis to identify interacting proteins.

### 2.7 Statistical Analysis

Statistical analyses were conducted using SPSS 23.0 (IBM Corp., NY, USA) and Graphpad Prism 10.0 (GraphPad Software Inc., CA, USA). Experimental data represented as the mean ± standard deviation (SD). For parametric data with equal variances, Student's *t*‐test (two groups) or one‐way ANOVA (three or more groups) was used to analyse the differences among groups. A *p*‐value < 0.05 was considered statistically significant.

## 3 Results

### 3.1 Activation of β3‐AR Regulates the Differential Gene Expression Profile and Pathways in Cardiac Fibroblasts

**FIGURE 1:**
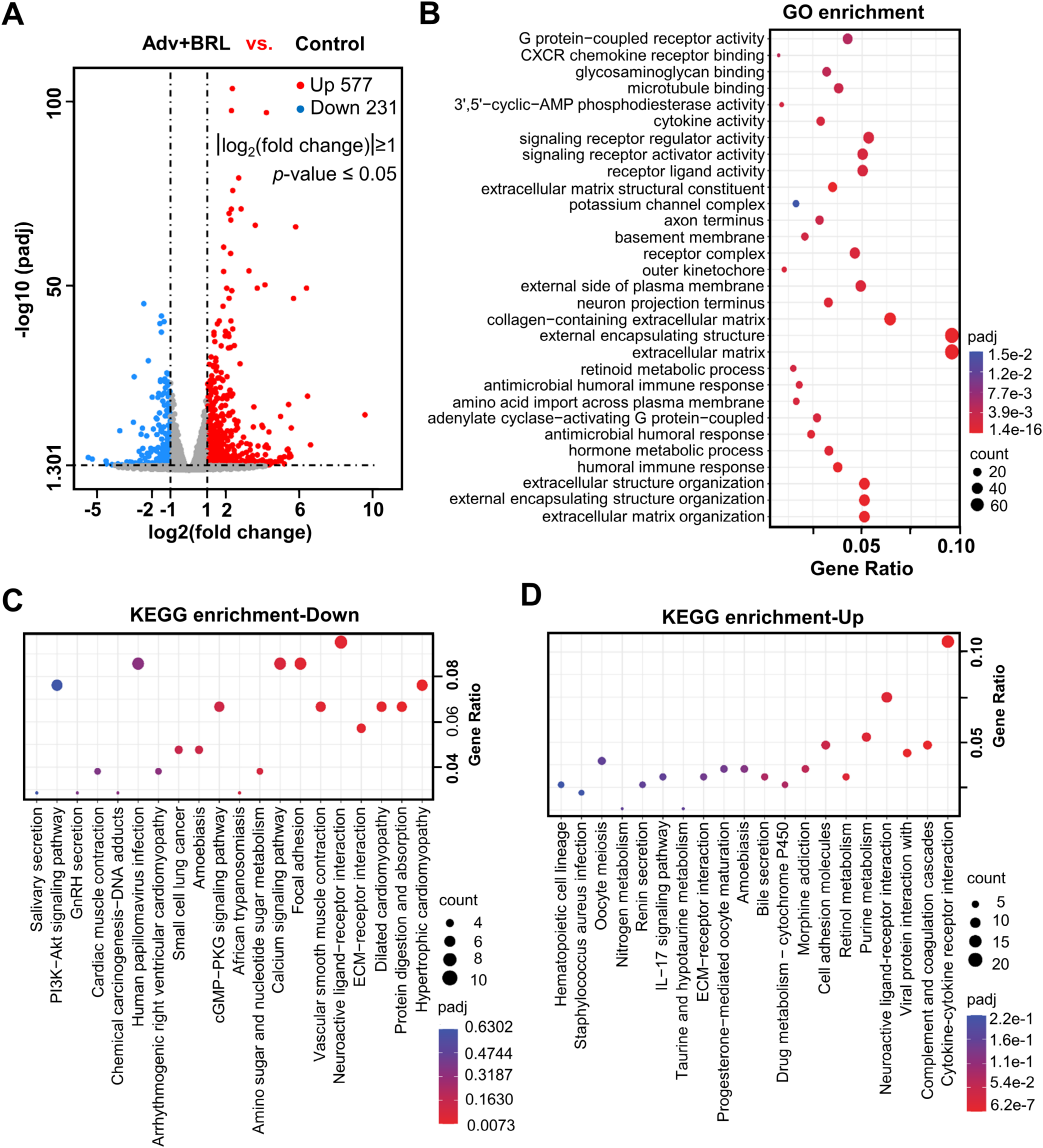
Activation of β3‐AR induced changes in the global gene expression profiles of neonatal mouse cardiac fibroblasts (NMCFs). (A) Volcano plot of upregulated (red) or downregulated (blue) transcripts in the Adv + BRL‐treated NMCFs vs. Control; *n* = 3. (B) Gene Ontology (GO) enrichment analysis of 908 differentially expressed genes (DEGs) (Fold Change ≥ 2 or ≤ −2, *p* < 0.05) between the Adv + BRL‐treated NMCFs and Control groups. The 30 most significantly enriched pathways are shown. (C) KEGG (Kyoto Encyclopedia of Genes and Genomes) pathway enrichment analysis of the identified down‐regulated DEGs between control and Adv + BRL‐treated NMCFs. (D) KEGG pathway enrichment analysis of the identified up‐regulated DEGs between control and Adv + BRL‐treated NMCFs. Adv: Adv‐Adrb3 (β3‐AR overexpression); BRL: BRL37344 (β3‐AR agonist).

To explore the role of β3‐AR in cardiac fibrosis, we first isolated fibroblasts from neonatal mouse heart tissues. Immunofluorescence identification confirmed normal fluorescence intensity of the fibroblast marker Vimentin (Figure S1), and the proportion of Vimentin‐positive cells with green fluorescence accounted for 93.62% of total counted cells, indicating relatively high purity of isolated fibroblasts that are suitable for subsequent cellular experiments. NMCFs were then infected with Adv‐Adrb3 overexpression lentivirus; immunofluorescence results showed optimal infection efficiency at a multiplicity of infection (MOI) = 50 (Figure S2), which was used for all subsequent experiments. After activation of β3‐AR‐overexpressing fibroblasts with the agonist BRL37344 (10 μM), RNA sequencing analysis (with |log₂(fold change)| ≥ 1 and *p* ≤ 0.05 as screening criteria) identified 577 upregulated genes and 231 downregulated genes (Figure 1A). Functional enrichment analysis of these differential genes revealed enrichment in “G protein‐coupled receptor activity”, “CXCR chemokine receptor binding”, “cytokine activity”, “extracellular matrix organisation”, and “collagen‐containing extracellular matrix” (Figure 1B), suggesting significant activation of G protein‐coupled receptor‐mediated extracellular matrix assembly—consistent with β3‐AR activation exacerbating cellular fibrosis. KEGG pathway enrichment analysis showed that downregulated genes were mainly enriched in pathways related to “cardiac muscle contraction”, “calcium signalling pathway”, “vascular smooth muscle contraction”, “extracellular matrix‐receptor interaction”, “dilated cardiomyopathy”, and “hypertrophic cardiomyopathy” (Figure 1C); upregulated genes were primarily enriched in inflammation and fibrosis‐related pathways such as “IL‐17 signalling pathway”, “cell adhesion molecules”, “neuroactive ligand‐receptor interaction”, and “cytokine‐cytokine receptor interaction” (Figure 1D). These results indicate that β3‐AR activation is closely associated with myocardial contraction, extracellular matrix deposition, and fibrosis, providing precise directions and a basis for subsequent exploration of β3‐AR activation mechanisms and key regulatory targets.

### 3.2 Inhibition of the JNK Pathway Alleviates β3‐AR Activation‐Induced Fibrosis

**FIGURE 2:**
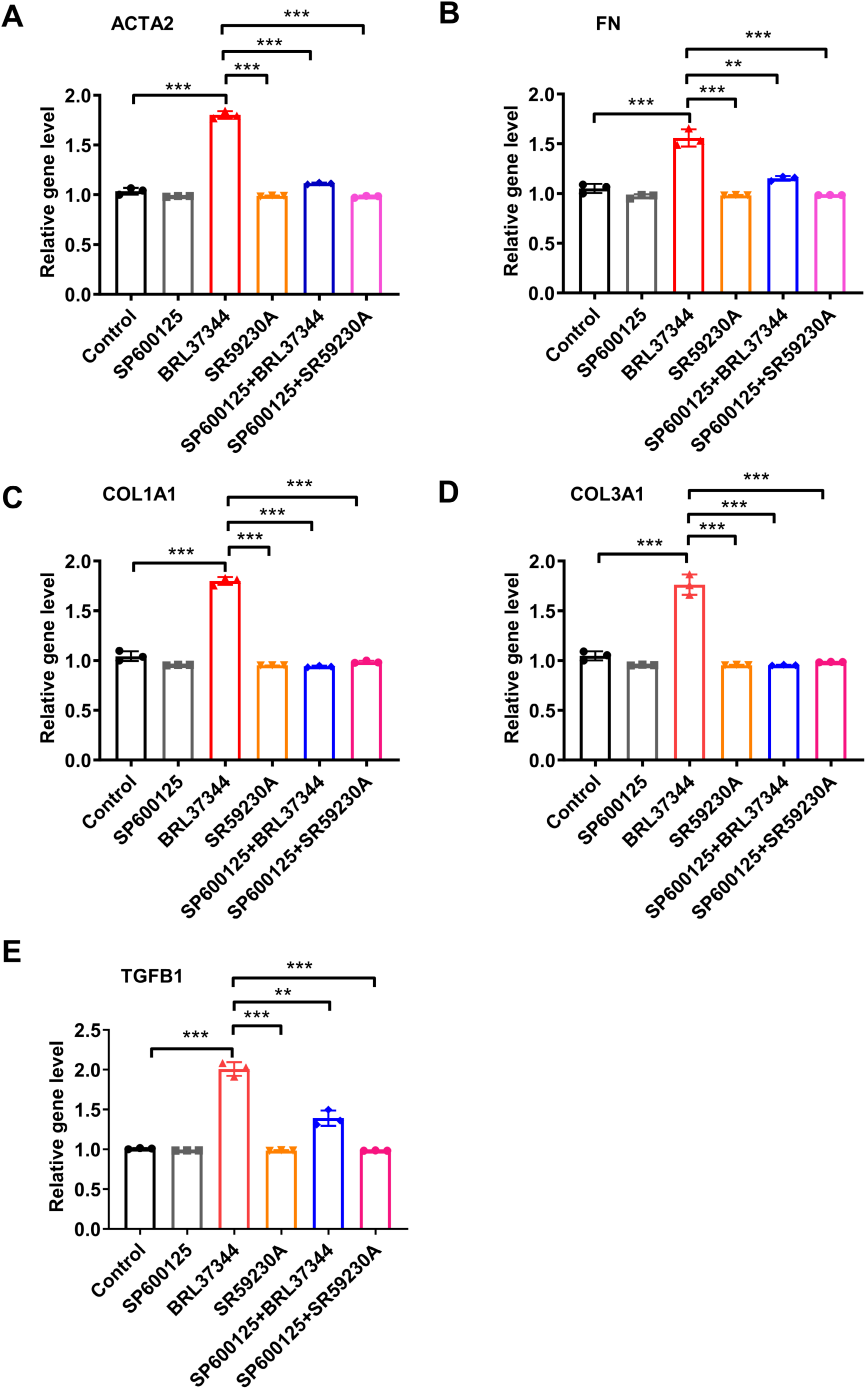
Activation of β3‐AR induced changes in cardiac fibrosis‐related gene expression of NMCFs. Real‐time PCR analysis of α‐SMA (ACTA2, A), fibronectin (FN, B), collagen I (COL1A1, C), collagen III (COL3A1, D), and TGF‐β1 (TGFB1, E) expression in NMCFs treated with β3‐AR overexpression and BRL37344 (β3‐AR agonist), or SR59230A (β3‐AR inhibitor), or SP600125 (JNK inhibitor) for 72 h. Data are represented as mean ± SD, *n* = 3; **p < 0.01, ***p < 0.001.

KEGG enrichment pathway analysis of RNA sequencing data showed significant enrichment of the cGMP‐PKG signalling pathway among downregulated genes in β3‐AR‐overexpressing and activated cells, consistent with previous reports [28, 29]. Additionally, our previous studies demonstrated that β3‐AR promotes cardiac fibrosis through the PKG/JNK/c‐Jun pathway [21]. We further investigated the role and mechanism of the JNK pathway in β3‐AR activation‐induced fibrosis in cardiac fibroblasts. Cardiac fibroblasts were treated with the β3‐AR agonist BRL37344 (10 μM), β3‐AR antagonist SR59230A (10 μM), and JNK inhibitor SP600125 (10 μM), and changes in mRNA expression of fibrosis markers α‐SMA (ACTA2), FN (Fibronectin), collagen I (COL1A1), collagen III (COL3A1), and TGF‐β1 (TGFB1) were measured. As shown in Figure 2, BRL37344 treatment significantly increased α‐SMA mRNA levels compared to the control group (*p* < 0.001). Treatment with SR59230A or SP600125 alone had no significant effect on α‐SMA mRNA levels; however, SP600125 significantly reduced BRL37344‐induced elevation of ACTA2 mRNA (*p* < 0.001), similar to the inhibitory effect of SR59230A (*p* < 0.001, Figure 2A). Consistent trends were observed for other fibrosis markers [30]: FN, COL1A1, and COL3A1 mRNA levels were significantly increased by BRL37344 but significantly reduced by SP600125 (Figure 2B–D). Similarly, BRL37344 significantly increased mRNA levels of the pro‐fibrotic upstream cytokine TGFB1 (*p* < 0.001), an effect reversed by SP600125 (*p* < 0.001) (Figure 2E). These results suggest that inhibition of the JNK pathway attenuates β3‐AR activation‐induced fibrosis.

**FIGURE 3:**
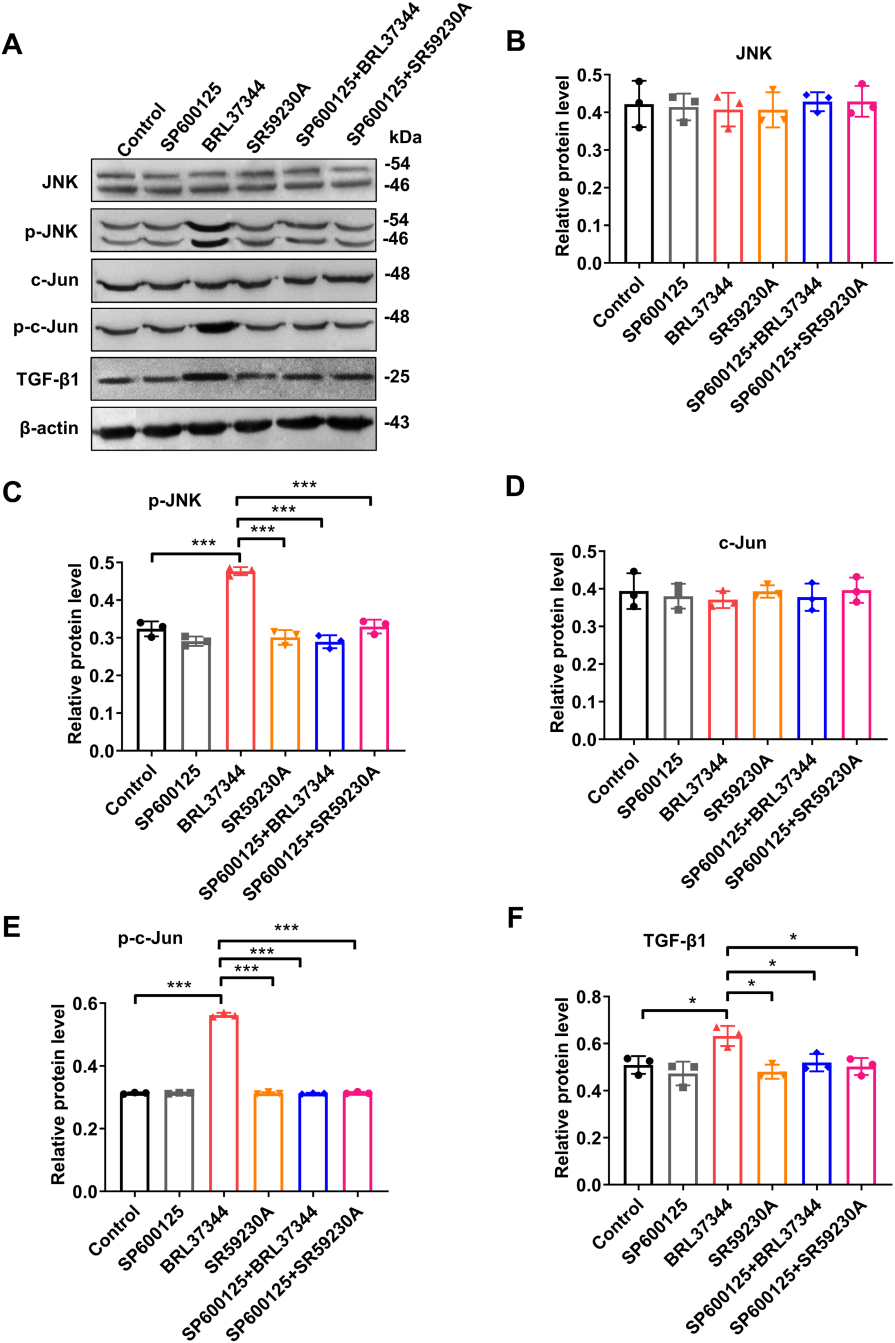
Activation of β3‐AR induced the activation of the JNK/c‐Jun signalling pathway and TGF‐β1 expression in NMCFs. (A) Western blot analysis of c‐Jun, p‐c‐Jun, JNK, p‐JNK, and TGF‐β1 protein expression in NMCFs treated with β3‐AR overexpression and BRL37344 (β3‐AR agonist), or SR59230A (β3‐AR inhibitor), or SP600125 (JNK inhibitor) for 72 h. Quantitative statistics of JNK (B), p‐JNK (C), c‐Jun (D), p‐c‐Jun (E), and TGF‐β1 (F) protein expression in NMCFs after β3‐AR overexpression and β3‐AR agonist or β3‐AR inhibitor treatment by Western blot analysis. Data are represented as mean ± SD, *n* = 3; *p < 0.05, ***p < 0.001.

To further evaluate the role of JNK and its related pathways in β3‐AR activation‐induced fibrosis, Western blotting was used to analyse protein level changes of JNK/c‐Jun/TGF‐β1 signalling molecules after β3‐AR activation. As shown in Figure 3A, while BRL37344 had no significant effect on total JNK protein levels (Figure 3B), it significantly upregulated JNK phosphorylation (p‐JNK) (*p* < 0.001, Figure 3C). BRL37344‐induced p‐JNK upregulation was abrogated by SP600125, but SP600125 had no significant inhibitory effect on JNK phosphorylation in SR59230A‐treated cardiac fibroblasts. Similar trends were observed for downstream c‐Jun and TGF‐β1: BRL37344 did not alter total c‐Jun protein levels (Figure 3D) but significantly upregulated phosphorylated c‐Jun (p‐c‐Jun) (*p* < 0.001, Figure 3E) and TGF‐β1 protein levels (*p* < 0.001, Figure 3F). These results confirm that the JNK pathway and its downstream c‐Jun/TGF‐β1 signalling play critical roles in β3‐AR activation‐induced fibrosis.

### 3.3 PTX Inhibition of Gi Signalling Does Not Prevent β3‐AR‐Induced Fibrotic Pathways

**FIGURE 4:**
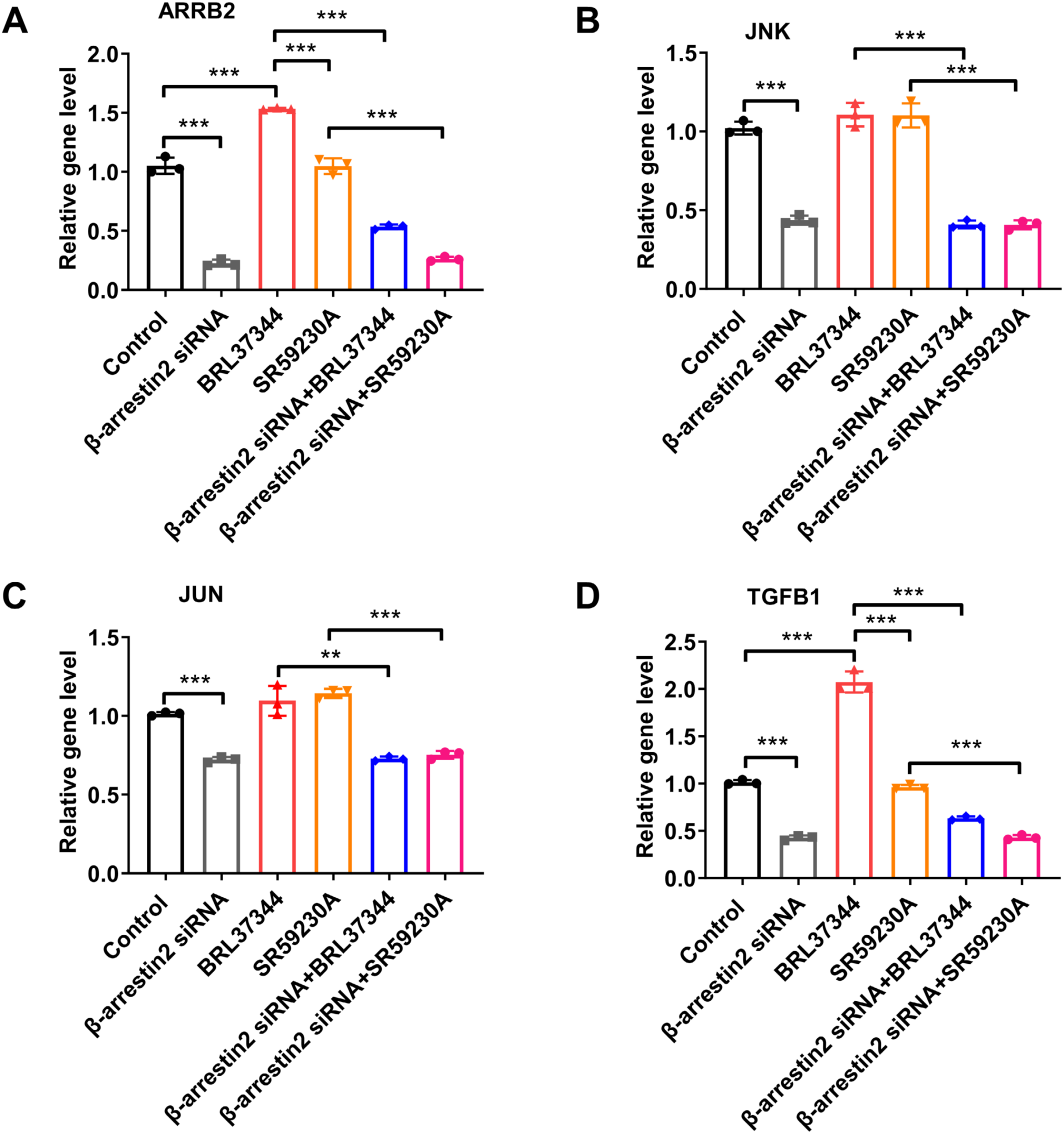
β‐arrestin2‐mediated β3‐AR‐activation‐induced TGF‐β1 expression. Real‐time PCR analysis of β‐arrestin2 (ARRB2, A), JNK (B), c‐Jun (JUN, C), and TGF‐β1 (TGFB1, D) expression in NMCFs transfected with β‐arrestin2 siRNA and β3‐AR overexpression and BRL37344 (β3‐AR agonist), or SR59230A (β3‐AR inhibitor) treatment. Data are represented as mean ± SD, *n* = 3; **p < 0.01, ***p < 0.001.

Previous studies have shown that β3‐AR exerts cardiac effects through Gi and its downstream signalling pathways. To investigate whether β3‐AR‐induced MF is associated with Gi signalling, we first inhibited Gi activity with PTX. Considering that β2‐AR can couple with inhibitory G proteins (Gi) and participate in cardiac rhythm regulation and myocardial remodelling, we simultaneously inhibited Gi activity and intervened in β‐arrestin2 expression in β3‐AR‐overexpressing cells, followed by treatment with β3‐AR‐specific agonist and antagonist to detect changes in JNK, c‐Jun, and TGF‐β1. As shown in Figure 4A, siRNA‐mediated knockdown of β‐arrestin2 (ARRB2) significantly downregulated its expression. Even after Gi inhibition by PTX, BRL37344 still upregulated β‐arrestin2 expression. β‐arrestin2 knockdown attenuated BRL37344‐induced β‐arrestin2 upregulation, though levels remained higher than in the siRNA‐only group; combined SR59230A treatment and β‐arrestin2 knockdown significantly reduced β‐arrestin2 expression compared to SR59230A alone, consistent with the siRNA‐only group. Additionally, after Gi inhibition by PTX, β‐arrestin2 knockdown downregulated mRNA levels of JNK (Figure 4B), JUN (c‐Jun) (Figure 4C), and TGFB1 (Figure 4D); despite Gi inhibition, BRL37344 still upregulated TGF‐β1 expression, while JNK and JUN mRNA levels remained unchanged—consistent with β3‐AR activation exerting effects through JNK and c‐Jun phosphorylation. β‐arrestin2 knockdown significantly downregulated JNK (Figure 4B) and JUN (Figure 4C) mRNA levels compared to the BRL37344 group; BRL37344‐induced TGF‐β1 upregulation was also reduced by siRNA interference (Figure 4D). Combined β‐arrestin2 siRNA and SR59230A treatment similarly significantly reduced JNK, JUN, and TGFB1 mRNA levels compared to SR59230A alone. These results indicate that β3‐AR activation can still alter downstream signalling pathways despite Gi inhibition, with β‐arrestin2 playing a crucial role in this process.

**FIGURE 5:**
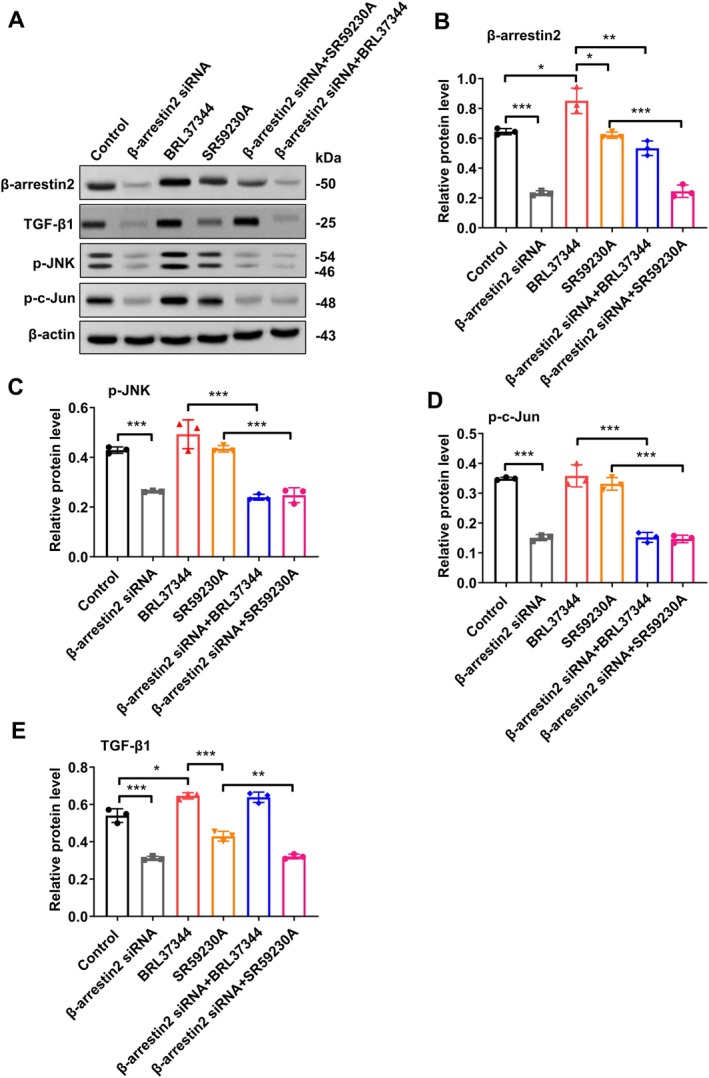
β3‐AR induced TGF‐β1 expression via upregulating β‐arrestin2 in NMCFs. (A) Western blot analysis of β‐arrestin2, JNK, c‐Jun, and TGF‐β1 expression in NMCFs transfected with β‐arrestin2 siRNA and β3‐AR overexpression and BRL37344 (β3‐AR agonist) or SR59230A (β3‐AR inhibitor) treatment. Quantitative statistics of β‐arrestin2 (B), JNK (C), c‐Jun (D), and TGF‐β1 (E) protein expression in NMCFs after β3‐AR overexpression and β3‐AR agonist or β3‐AR inhibitor treatment by Western blot analysis. Data are represented as mean ± SD, *n* = 3; *p < 0.05, **p < 0.01, ***p < 0.001.

Consistent with PCR results, Western blotting was used to detect protein levels of β‐arrestin2, JNK, c‐Jun, and TGF‐β1 after Gi inhibition. As shown in Figure 5A,B, BRL37344 still upregulated β‐arrestin2 protein levels in PTX‐pretreated NMCFs (*p* < 0.05), while siRNA intervention significantly reduced β‐arrestin2 protein levels (*p* < 0.001) and attenuated BRL37344‐induced upregulation (*p* < 0.01). Although SR59230A did not significantly reduce β‐arrestin2 protein levels under Gi‐inhibited conditions, combined siRNA intervention resulted in a significant decrease (*p* < 0.001 vs. SR59230A). These results confirm that β‐arrestin2 plays an important role in β3‐AR‐mediated pro‐fibrotic effects. Similar trends were observed for JNK, c‐Jun, and TGF‐β1 protein levels: After Gi inhibition, BRL37344 did not significantly upregulate JNK (Figure 5A,C) or c‐Jun (Figure 5A,D) protein levels but significantly increased TGF‐β1 protein levels (Figure 5A,E, *p* < 0.05). β‐arrestin2 siRNA significantly reduced protein levels of JNK (*p* < 0.001), c‐Jun (*p* < 0.001), and TGF‐β1 (*p* < 0.001) (Figure 5A,C–E) and attenuated BRL37344‐induced upregulation of these proteins (*p* < 0.001). Similar to the β3‐AR agonist, SR59230A did not significantly downregulate JNK or c‐Jun protein levels, but combined siRNA interference resulted in significant reductions in JNK, c‐Jun, and TGF‐β1 protein levels. These results suggest that cardiac β3‐AR regulates MF through β‐arrestin2‐mediated biased activation of the JNK/c‐Jun pathway.

### 3.4 β3‐AR Promotes Fibrosis Through Its Interaction With β‐arrestin2

**FIGURE 6:**
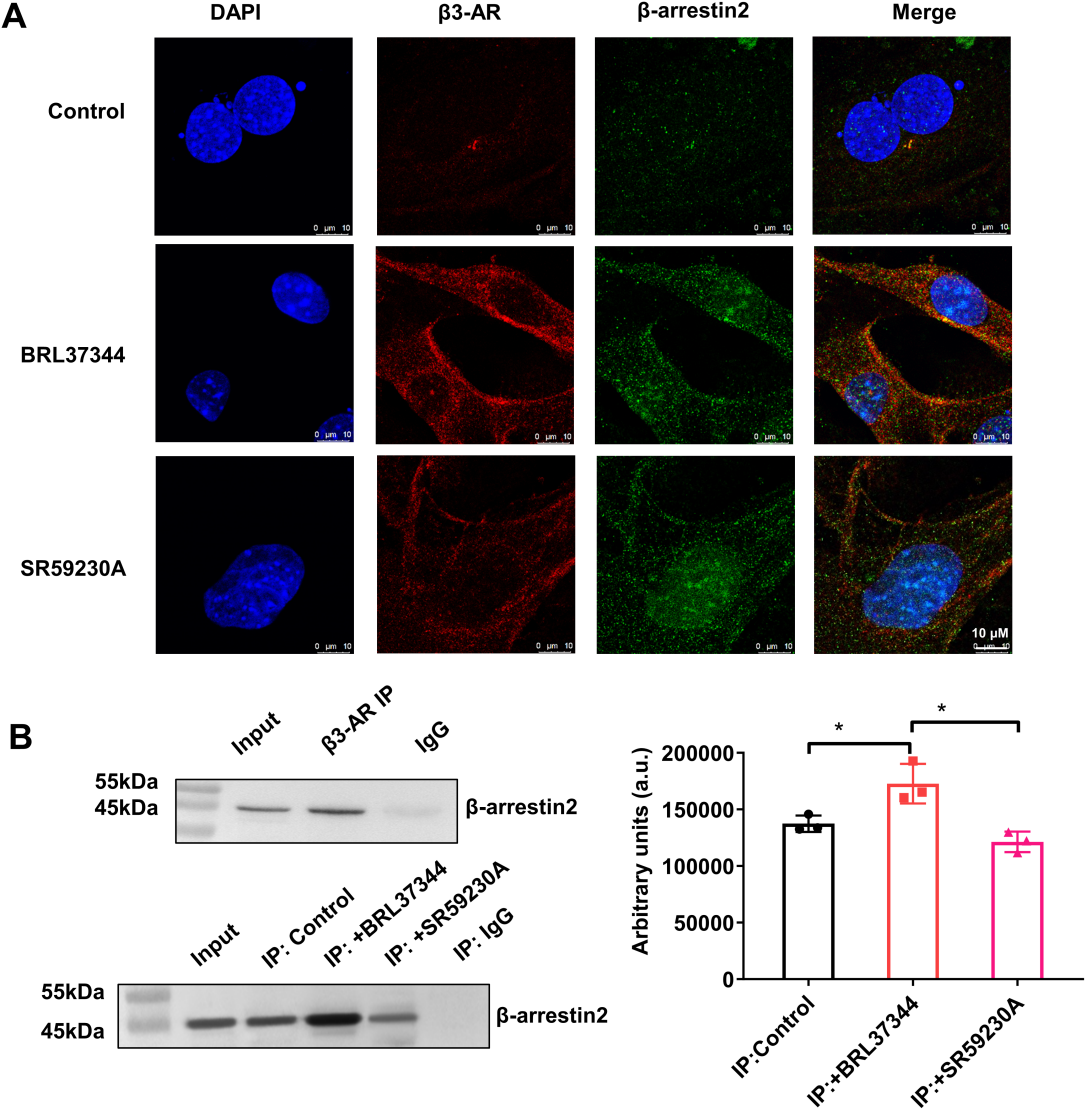
β3‐AR regulates cardiac fibrosis via interacting with β‐arrestin2. (A) Immunofluorescence colocalisation of β3‐AR (red) and β‐arrestin2 (green) in NMCFs transfected with β3‐AR overexpression and BRL37344 (β3‐AR agonist), or SR59230A (β3‐AR inhibitor) treatment. Scale bars = 10 μm. (B) Immunoprecipitation was performed to detect interactions between β3‐AR and β‐arrestin2 in NMCFs after β3‐AR overexpression and β3‐AR agonist or β3‐AR inhibitor treatment. *n* = 3. Data are represented as mean ± SD, *n* = 3; *p < 0.05.

To investigate the role of β‐arrestin2 in β3‐AR‐mediated biased activation of MF, immunofluorescence was first used to examine the cellular localisation of β3‐AR and β‐arrestin2. As shown in Figure 6A, β3‐AR overexpression and activation resulted in significant colocalisation of β3‐AR and β‐arrestin2. Inhibition of β3‐AR activity with SR59230A attenuated this colocalisation signal, suggesting that β3‐AR expression and activity may recruit β‐arrestin2 to exert effects. To further verify this hypothesis, coimmunoprecipitation (Co‐IP) was used to examine the interaction between β3‐AR and β‐arrestin2. Co‐IP results from NMCFs showed that β‐arrestin2 could be immunoprecipitated with a β3‐AR antibody (Figure 6B). Additional treatment with BRL37344 (to activate β3‐AR) or SR59230A (to inhibit β3‐AR) revealed that BRL37344 enhanced the interaction between β3‐AR and β‐arrestin2 (Figure 6B). These results demonstrate that β3‐AR and β‐arrestin2 colocalise and interact significantly; β3‐AR promotes MF through interaction with β‐arrestin2, thereby activating the downstream JNK/c‐Jun pathway and increasing secretion of the pro‐fibrotic cytokine TGF‐β1.

## 4 Discussion

**FIGURE 7:**
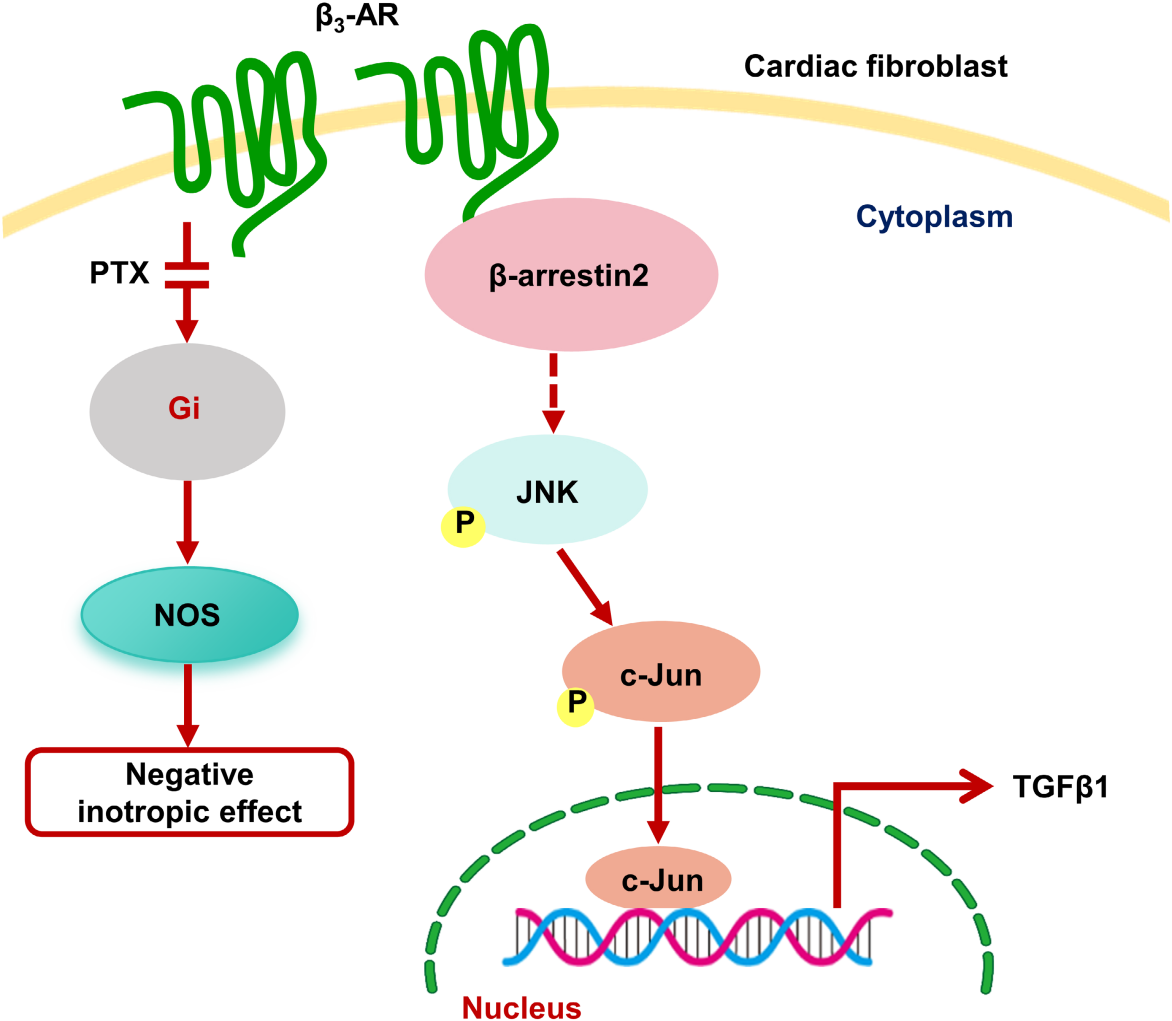
Schematic diagram depicting the mechanism of β3‐AR regulatory pathway in cardiac fibrosis. β3‐AR interacts with β‐arrestin2 and activates the downstream JNK/c‐Jun signalling pathway, thereby upregulating the pro‐fibrotic cytokine TGF‐β1.

In this study, we overexpressed β3‐AR in NMCFs to simulate the upregulation of β3‐AR during myocardial remodelling. Immunofluorescence results showed enhanced fibroblast characteristics after β3‐AR overexpression; RNA sequencing suggested that β3‐AR‐mediated pro‐fibrotic effects may be associated with the β‐arrestin2/JNK/c‐Jun signalling pathway. Using a JNK inhibitor and β‐arrestin2 siRNA, we confirmed that β‐arrestin2 and the JNK/c‐Jun signalling pathway play critical roles in this process. Mechanistically, we found significant colocalisation and interaction between β3‐AR and β‐arrestin2; β3‐AR recruits β‐arrestin2 and activates the downstream JNK/c‐Jun signalling pathway, thereby upregulating the pro‐fibrotic cytokine TGF‐β1 (Figure 7). This study is the first to propose a β‐arrestin2‐mediated biased activation mode of β3‐AR, providing novel therapeutic targets for intervening in MF and treating HF with more precise strategies.

As a G protein‐coupled receptor (GPCR) with highly controversial functions in the cardiovascular system, the complexity of β3‐adrenergic receptor (β3‐AR) signalling mechanisms and cell‐specific effects has long hindered accurate understanding of its role in myocardial remodelling. As the only β‐AR subtype that is continuously upregulated and resistant to receptor desensitisation in HF, its classical function is thought to involve coupling with Gi proteins to activate the NOS/cGMP/PKG signalling axis, exerting a negative inotropic effect in cardiomyocytes to reduce myocardial oxygen consumption and counteract the damaging effects of excessive β1‐AR activation. However, this understanding is highly contradictory: Rozec et al. confirmed that cardiac‐specific overexpression of β3‐AR inhibits MF [31], while Sheng et al. found that β3‐AR upregulation exacerbates atrial fibrosis and electrical remodelling in a rapid pacing‐induced atrial fibrillation model [32]. Our team's previous studies also suggested that β3‐AR may mediate cardiac fibrosis through the TGF‐β/Smad pathway. More complexly, Gao Pingjin's team demonstrated that the β3‐AR agonist mirabegron exerts protective effects by promoting lymphangiogenesis in an aortic dissection/aneurysm model [33], highlighting the tissue‐specific nature of its functions.

The core of this controversy lies in the diversity of β3‐AR signal transduction—beyond the traditional G protein‐dependent pathway, β‐arrestin‐mediated non‐G protein pathways have been confirmed as key nodes of GPCR‐biased activation [34]. However, few studies have explored the role and mechanism of β3‐AR in cardiac fibroblasts, the core effector cells of fibrosis. MF is driven by fibroblast activation and collagen deposition, with sympathetic nerve activation as a key trigger. As the only upregulated β‐AR subtype in HF, the expression changes and functional effects of β3‐AR in fibroblasts remain unclear—this constitutes the core motivation for focusing on NMCFs and exploring the mechanisms of β3‐AR‐regulated fibrosis in this study.

By overexpressing β3‐AR to simulate the pathological upregulation of the receptor in HF, this study is the first to confirm a clear pro‐fibrotic effect of β3‐AR activation on cardiac fibroblasts in vitro. Results showed that the β3‐AR‐specific agonist BRL37344 significantly upregulated mRNA expression of fibrosis markers such as α‐SMA, FN, and type I/III collagen, while increasing TGF‐β1 levels; the JNK inhibitor SP600125 completely reversed these effects, indicating that the JNK pathway is a key downstream mediator. This finding aligns with mechanistic observations in other disease areas: In liver cirrhosis models, β3‐AR agonists regulate tissue remodelling through receptor‐mediated signalling pathways, with effects dependent on interactions between specific signalling molecules [35]. Through RNA sequencing and functional validation, this study further locked the mechanism onto the β‐arrestin2/JNK/c‐Jun signalling axis, complementing the complete regulatory chain of “receptor‐adaptor‐kinase‐cytokine”—consistent with the known function of β‐arrestin2 as a GPCR signalling scaffold protein that can recruit and activate MAPK kinases such as JNK [36]. Myocardial fibrosis progresses via concomitant fibroblast proliferation and myofibroblast transformation [37]. Existing data confirm β‐arrestin2‐dependent JNK cascade drives cardiac fibroblast proliferation upon GPCR activation [23], and pathological β3‐AR upregulation promotes persistent fibroblast overproliferation in failing hearts. Thus, interrupting β3‐AR/β‐arrestin2 interaction confers dual benefits to restrain both fibroblast proliferation and ECM deposition.

Notably, through Gi protein inhibition experiments, this study revealed a unique signal transduction mode of β3‐AR in cardiac fibroblasts—its pro‐fibrotic effect is independent of the classical Gi protein pathway and relies on β‐arrestin2‐mediated biased activation. Traditional views hold that β3‐AR in the heart primarily transmits signals through Gi proteins [38]; however, after inhibiting Gi activity with pertussis toxin, BRL37344 still upregulated TGF‐β1 expression and activated the JNK/c‐Jun pathway. In contrast, siRNA‐mediated β‐arrestin2 knockdown significantly blocked these effects, and immunofluorescence confirmed that β3‐AR activation induces colocalisation with β‐arrestin2 and promotes its nuclear translocation. This result challenges the traditional understanding of β3‐AR signalling and aligns with the theory that “GPCRs can exert specific effects through β‐arrestin‐mediated non‐G protein pathways” [39]. It also provides a new perspective for explaining functional controversies: β3‐AR may exert protective effects through the Gi pathway in cardiomyocytes, while promoting fibrosis through the β‐arrestin2 pathway in fibroblasts.

However, this study has several limitations. First, the β3‐AR overexpression model: To simulate pathological receptor upregulation, this study used a lentiviral overexpression strategy, resulting in receptor distribution in both the cell membrane and cytoplasm—consistent with the physiological localisation primarily in the cell membrane [40]—which may affect the authenticity of signal transduction. Second, the limitations of in vitro experiments: The study was conducted only on neonatal mouse fibroblast cultures, lacking validation in in vivo models such as myocardial infarction or pressure overload, and cannot reflect the influence of complex microenvironments such as inflammatory factors and cell–cell interactions in vivo. Gao Pingjin's team has confirmed that adipose‐lymphatic vessel interactions in the in vivo microenvironment significantly regulate β3‐AR functional effects [33]. Third, insufficient depth of mechanistic research: While the interaction between β3‐AR and β‐arrestin2 was confirmed, the specific binding domains were not identified, and the involvement of molecules such as GRK in receptor phosphorylation and β‐arrestin2 recruitment was not explored [41]. Specifically, accumulated biochemical evidence highlights GRK2 as the predominant kinase governing β3‐AR desensitisation and subsequent β‐arrestin2 recruitment via its unique RGS‐homology domain [8], and Ser247 at the C‐terminal intracellular tail of β3‐AR has been validated as a critical phosphorylation residue modulating receptor conformation and β‐arrestin binding capability [42]. In general, GRK‐mediated sequential multi‐site phosphorylation constitutes the “phosphorylation barcode” dictating GPCR‐biased signalling switching between G‐protein and β‐arrestin cascades [43]. Site‐directed mutagenesis focusing on Ser247 and other potential GRK‐targeted serine/threonine residues will be implemented in our follow‐up study to pinpoint the exact docking motifs for β‐arrestin2 recruitment onto activated β3‐AR. Additionally, the role of the TGF‐β1‐downstream Smad pathway was not established. As our earlier work demonstrated that TGFβ/Smad is involved in cardiomyocyte proliferation [44], the regulatory mechanism of this pathway remains unclear. Future studies should construct fibroblast‐specific β‐arrestin2 knockout mice to investigate whether β3‐AR promotes cardiac fibrosis through binding to β‐arrestin2.

In conclusion, this study is the first to clarify a novel mechanism by which β3‐AR promotes fibrosis in cardiac fibroblasts through the β‐arrestin2/JNK/c‐Jun/TGF‐β1 pathway, proposing that “cell‐specific signal bias” is a key driver of its functional controversy and filling the gap in research on β3‐AR in fibroblasts. This study expands the understanding of β3‐AR non‐G protein signalling and provides a target for developing drugs targeting β‐arrestin2 interactions—blocking the β3‐AR/β‐arrestin2 interaction may inhibit fibrosis while preserving myocardial protective effects.

## Author Contributions

**Yang Dong:** methodology, validation, visualization, formal analysis. **Zhongcheng Xu:** conceptualization, methodology, data curation, investigation, visualization, funding acquisition, writing – original draft, writing – review and editing, formal analysis. **Jun Liu:** data curation, methodology, formal analysis, visualization. **Juncang Duan:** methodology, data curation, formal analysis, validation. **Jian Ding:** methodology, formal analysis, validation. **Chun Zhou:** methodology, formal analysis, validation, data curation. **Jie Wu:** data curation, validation, formal analysis. **Min Zhu:** conceptualization, methodology, data curation, investigation, formal analysis, visualization, writing – original draft, writing – review and editing. **Xuan Ying:** data curation, formal analysis, validation, visualization, software. **Zhaohui Qiu:** conceptualization, funding acquisition, investigation, supervision, resources, project administration, writing – review and editing. **Wenbiao Wang:** investigation, project administration, funding acquisition, supervision, resources, writing – review and editing, conceptualization.

## Funding

This work was supported by grants from Zhejiang Provincial Natural Science Foundation of China [LQ21H020002], Scientific Research Fund Project of Shanghai Sixth People's Hospital Medical Group [ly202403], and the Fund for the Science and Technology research Plan of Jinhua City, China [2020‐3‐060].

## Conflicts of Interest

The authors declare no conflicts of interest.

## Supporting information


**Figure S1:** Immunofluorescence identification of cardiac fibroblasts using the fibroblast marker Vimentin. Scale bars = 25 μm. Original magnification × 200.
**Figure S2:** The fluorescence intensity of GFP in cardiac fibroblasts treated with different MOIs of Adv‐Adrb3 infection. Scale bars = 10 μm. Original magnification × 400.
**Table S1:** Primers used for quantitative RT‐PCR in this study.

## Data Availability

The data that support the findings of this study are available from the corresponding author upon reasonable request.
