# Supplementary material for: β3‐AR/β‐arrestin2 Interaction Triggers Cardiac Fibrosis Through JNK/c‐Jun Pathway in Cardiac Fibroblasts
Source: J Cell Mol Med. 2026 Aug 3;30(15):e71305. doi: 10.1111/jcmm.71305 (PMC13433835; doi:10.1111/jcmm.71305)
Supplement: Supplementary file 1 [file JCMM-30-e71305-s001.docx]

**Supplementary Figure 1**. Immunofluorescence identification for cardiac fibroblasts using the fibroblast marker Vimentin. Scale bars=25 μm. Original magnification × 200.

**Supplementary Figure 2**. The fluorescence intensity of GFP in cardiac fibroblasts treated with different MOIs of Adv-Adrb3 infection. Scale bars=10 μm. Original magnification × 400.

| **Supplementary Table 1.** Primers used for quantitative RT-PCR in this study | |
| --- | --- |
| **Primer name** | **Sequence (5’-3’)** |
| ACTA2-Forward | TGAAGAGCATCCGACACT |
| ACTA2-Reverse | GCCTGAATAGCCACATACA |
| ARRB2-Forward | GATACCAACTATGCCACAGA |
| ARRB2-Reverse | GTCATCACAGTCATCATCCT |
| TGFB1-Forward | ACTCTCCACCTGCAAGACC |
| TGFB1-Reverse | CTGGCGAGCCTTAGTTTGGAC |
| JUN-Forward | ATGACCTTGAACCTGGCCGAC |
| JUN-Reverse | TGCCCATTGCTGGACTGGAT |
| COL1A1-Forward | GAGAACATCCGCAGCCCCGAAG |
| COL1A1-Reverse | TCGATCCAGTACTCTCCGCTCT |
| COL3A1-Forward | AGTCTTATAAGCCCTGATGGT |
| COL3A1-Reverse | TCCATCTTGCAGCCTTGGTT |
| JNK-Forward | AATACGCTGGATATAGCTTT |
| JNK-Reverse | GGATAACAAATCTCTTGCCT |
| FN1-Forward | CAGTTCAGAGGAGCATCAG |
| FN1-Reverse | GGCATTGTCGTTCAGAGT |
| ACTB-Forward | CTCCTGAGCGCAAGTACTCT |
| ACTB-Reverse | TACTCCTGCTTGCTGATCCAC |
